# No effect of thymosin beta‐4 on the expression of the transcription factor Islet‐1 in the adult murine heart

**DOI:** 10.1002/prp2.407

**Published:** 2018-06-03

**Authors:** Florian Weinberger, Philipp Nicol, Jutta Starbatty, Mandy Stubbendorff, Peter M. Becher, Sonja Schrepfer, Thomas Eschenhagen

**Affiliations:** ^1^ Department of Experimental Pharmacology and Toxicology University Medical Center Hamburg‐Eppendorf Hamburg Germany; ^2^ DZHK, German Center for Cardiovascular Research partner site Hamburg/Kiel/ Lübeck Germany; ^3^ Department of Cardiovascular Surgery, Transplant and Stem Cell Immunobiology (TSI) Lab University Heart Center Hamburg Hamburg Germany; ^4^ Department of General and Interventional Cardiology University Heart Center Hamburg Hamburg Germany

**Keywords:** cardiac progenitor cells, cardiac regeneration, cardiac transcription factors, thymosin beta‐4

## Abstract

The transcription factor Islet‐1 marks a progenitor cell population of the second heart field during cardiogenesis. In the adult heart Islet‐1 expression is limited to the sinoatrial node, the ventricular outflow tract, and parasympathetic ganglia. The regenerative effect in the injured mouse ventricle of thymosin beta‐4 (TB4), a 43‐aminoacid peptide, was associated with increased Islet‐1 immunostaining, suggesting the induction of an Islet‐1‐positive progenitor state by TB4. Here we aimed to reassess this effect in a genetic model. Mice from the reporter mouse line Isl1‐nLacZ were primed with TB4 and subsequently underwent myocardial infarction. Islet‐1 expression was assessed 2, 7, and 14 days after infarction. We detected only a single Islet‐1^+^ cell in 8 TB4 treated and infarcted hearts which located outside of the sinoatrial node, the outflow tract or cardiac ganglia (in ~2500 sections). Two cells were identified in 5 control infarcted hearts. TB4 did not induce LacZ positivity in ventricular explants cultures of Isl1‐nLacZ mice nor did it affect the density of LacZ^+^ cells in explant cultures of nLacZ^+^ regions of the heart. In summary, we found no evidence that TB4 reactivates Islet‐1 expression in adult mouse ventricle.

**Abbreviations**
LAD: left anterior descending artery
SMA: smooth muscle actin

## 1 INTRODUCTION

Regenerative strategies might represent new therapeutic approaches to heart failure, which is still the leading cause of death in the western world.^1^ One possible treatment strategy aims at stimulating proliferation and differentiation of endogenous progenitor cells in the adult heart. Although several endogenous progenitor cell populations have been described, their exact physiological role and cardiogenic potential is still unknown or controversial.^2^ Islet‐1^+^ cells are a prominent example among the cells in question. During cardiac development Islet‐1 marks a progenitor cell population of the second heart field. These Islet‐1^+^ cells are capable of differentiating to cardiomyocytes, smooth muscle cells, and endothelial cells and contribute to the right ventricle, the right atrium and the outflow tract.^3^, ^4^, ^5^ Islet‐1 knock‐out leads to severe cardiac malformation,^6^ substantiating its crucial role for cardiac development. In contrast, Islet‐1 expression in the adult heart is limited to very distinct regions^7^, ^8^: cells of the outflow tract and parasympathetic ganglia as well as the sinoatrial node, for which an important functional role of Islet‐1 has recently been established.^9^, ^10^, ^11^, ^12^

Thymosin beta‐4 (TB4) is a 43‐aminoacid peptide which derives its name from the fact that it was first discovered in thymus. It is, however, expressed ubiquitously and present in most cells at high concentration.^13^ Initial studies focused on its role as an actin‐sequestering molecule that inhibits the polymerization of globular actin (G‐actin).^14^ More recently pleiotropic effects of TB4 have been described. Its role in cardiac development is controversial. While some reported that TB4 is essential for cardiac development and promoted cell survival, migration, and regeneration after cardiac injury,^15^, ^16^ others failed to observe a cardiac phenotype in TB4 knockout mice,^17^, ^18^ suggesting that it is not required for cardiac development. Since the initial description of its beneficial properties in myocardial injury^15^ several studies have evaluated TB4 in the context of myocardial injury. TB4 improved vasculogenesis, reduced infarct size, prevented ventricular rupture and improved left ventricular function after myocardial infarction. It also extended the time window for cardiac regeneration after birth and amplified the effect of Gata4, Mef2c, and Tbx5 to transdifferentiate fibroblasts to cardiomyocytes.^19^, ^20^, ^21^, ^22^, ^23^, ^24^, ^25^, ^26^

Administration of TB4 before myocardial injury activated epicardial progenitor cells (marked by the expression of Wilm's tumor 1 protein, WT1) and thereby led to de novo cardiomyogenesis.^27^ In this work, the epicardial progenitor cells in the infarct area stained positive for Islet‐1 shortly after injury. Given the limited specificity of immunolabeling in histological preparations we set out to reevaluate the effect of TB4 on distribution and role of Islet‐1^+^ cells after injury using a genetically defined model.

## 2 MATERIALS AND METHODS

### 2.1 Animals

The investigation conforms to the guide for the care and use of laboratory animals. This study was carried out in accordance with the recommendations of the NIH (Publication No. 85‐23, revised 1985). The protocol was approved by the local authorities (Behörde für Gesundheit und Verbraucherschutz‐Veterinärwesen/Lebensmittelsicherheit). Isl1‐nLacZ mice were generated and characterized as described previously.^3^ Investigations were done in male and female heterozygous Isl1‐nLacZ mice on a mixed Black Swiss and C57BL/6J background.

### 2.2 Experimental myocardial infarction

Myocardial infarction was induced by permanent ligation of the left anterior descending artery (LAD) as previously described.^28^ Mice were isoflurane‐anesthesized (2.5%) and mechanically ventilated. Animals were assured to a warming platform in a supine position. Buprenorphine (0.05 mg/kg body 86 weight) and carprofen (5 mg/kg body weight) were injected subcutaneously 15 minute prior to the operation. Left‐lateral thoracotomy was performed. The pericardium was opened and the LAD was ligated with a single stitch (8‐0 Prolene suture, Ethicon, Norderstedt, Germany). Analgetic treatment (buprenorphine 0.05 mg/kg body weight twice per day) and carprofen (5 mg/kg body weight/day) was continued 5 days postoperatively. Injection of TB4 (12 mg/kg bodyweight) was performed following a protocol from Smart et al.^27^ TB4 was injected daily for 7 days prior to LAD‐ligation and every other day after the surgery. Control animals were injected with PBS. Mice were sacrificed 2, 7, and 14 days after LAD‐ligation by cervical dislocation. Hearts were then perfusion fixed with 4%formaldehyde in situ, harvested and washed in 0.9% NaCl.

### 2.3 Bromo‐chloro‐indolyl‐galactopyranoside staining

Hearts were incubated in a staining solution containing potassium ferrocyanide (5 mmol/L), potassium ferricyanide (5 mmol/L), MgCl_2_ (2 mmol/L), 0.02% NP‐40, 0.01% sodium deoxycholate, Tris pH 7.4 (20 mmol/L), and 1 mg/mL bromo‐chloro‐ indolyl‐galactopyranoside (X‐Gal) in phosphate‐buffered saline for 2 hours at 37°C. Cells were fixed in 0.05% glutaraldehyde for 10 minutes at 4°C and subsequently incubated in the staining solution over night. Islet‐1^+^ cells could be visualized by a blue nuclear signal.

### 2.4 Histology

Hearts were embedded in a sucrose (30%)/Tissue‐Tek (Sakura) mixture (1:1) and serial cryosections (10 μm) of the heart were acquired in a transversal (short) axis. All sections were collected on Superfrost Plus‐slides (Fisher Scientific). Hematoxylin and eosin staining (H&E) was performed to allow for an overview and Picro‐Sirius red staining was performed to analyze infarct size. Consecutive sections were used for immunofluorescence staining. X‐Gal^+^ structures were identified with an x10 objective. These structures were further analyzed in higher magnification with an x40 objective. Cells showing a clear blue nuclear signal and a costaining with DRAQ5 were counted (nLacZ^+^). Pictures were taken with an Axio Scope 2 microscope driven by the Axiovison software (Zeiss).

### 2.5 Immunostaining

Sections and cells were blocked in a solution containing TBS pH 7.4 (50 mmol/L), FCS (10%), BSA (1%), Triton X‐100 (0.5%) for 1 hour. Immunostaining was performed at 4°C over night for the first antibody and at room temperature for 1‐2 hours for the second antibody. Antibodies were used as follows: α‐actinin 1:800 (Sigma‐Aldrich A 7811), DDR2 1:800 (SantaCruzBiotechnology, Inc., sc‐7555), smooth muscle actin 1:800 (R&D Systems, Clone 1A4), Nkx 2.5 1:100 (Santa Cruz Biotechnology, Inc., sc‐14033), WT1 1:250 (Abcam, ab89901), Alexa fluor^®^ 488 1:800 (Goat anti mouse 2 mg/mL, Molecular Probes A 11017), Alexa fluor^®^ 488 1:800 (Goat anti rabbit 2 mg/mL, Molecular Probes A 11034) in a solution containing TBS 0.05 mol/L, pH 7.4, 1% BSA, 0.5% Triton X‐100. DRAQ5 1:1000 (Biostatus limited BOS‐889‐001‐R050) was used to stain nuclei. Confocal laser scanning microscopy was performed with a Zeiss LSM 510 META system.

### 2.6 Infarct size measurement

Sections from infarcted hearts were stained with Picrosirius red and examined with a Zeiss Axiovert 25 microscope using a x1.25 objective. Images were captured with a Jenoptik ProgRes SpeedXT core 5 camera driven by the ProgRes Capture Pro software. ImageJ 1.8.0 software was used to measure scar and left ventricular myocardial areas. The scar was measured by an investigator who was blinded to the identity of the sections. Infarct scar area and total area of the left ventricular myocardium from 7 to 8 sections per heart were traced manually and measured automatically. Infarct size was calculated by dividing the sum of infarct areas by the sum of LV areas from all sections (including those without infarct scar) and multiplying by 100 (expressed in percentage with SEM).

### 2.7 Cell cultures

Cell culture conditions were adapted from Smart and Riley.^29^ Hearts were harvested from adult Isl1‐nLacZ mice, minced and placed on cell culture dishes under sterile conditions. The cultures were then incubated up to 14 days at 37°C in DMEM, fetal calf serum (FCS, 15%), PenStrep (1%) and TB4 (100 ng/mL). Culture medium did not contain a TGF‐β inhibitor.^29^ Control cell cultures contained medium without TB4.

## 3 RESULTS

### 3.1 TB4 effects in vivo

**Figure 1:**
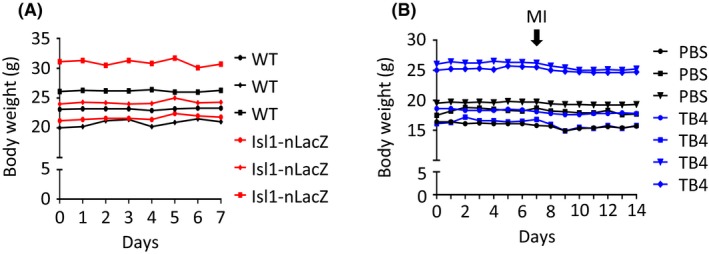
Body weight after TB4 treatment. (A) Body weight in healthy WT and Isl1‐nLacZ animals after TB4 treatment. TB4 (12 mg/kg body weight, i.p.) was administered daily for 7 days. (B) Body weight in infarcted Isl1‐nLacZ animals. PBS or TB4 (12 mg/kg body weight, i.p) were administered daily for 7 days prior to LAD ligation and every other day after myocardial infarction

**Figure 2:**
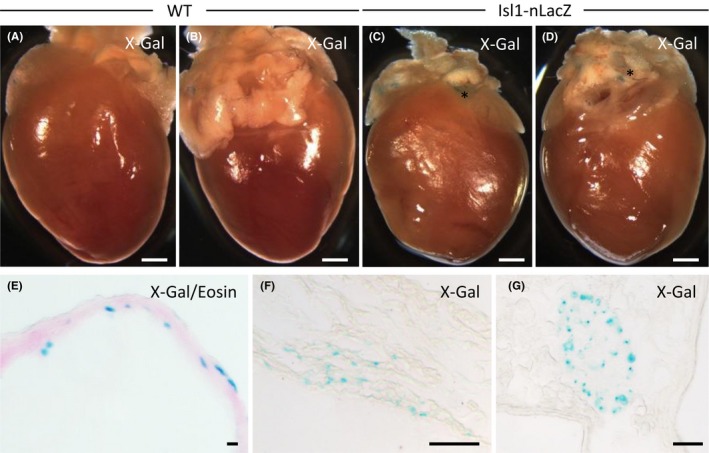
Hearts from healthy TB4 primed WT and Isl1‐nLacZ animals. (A and B) Anterior and posterior view of an X‐Gal stained TB4 primed WT heart. (C and D) Anterior and posterior view of a TB4 primed Isl1‐nLacZ animal after X‐Gal staining. X‐Gal‐positive areas are marked with an asterisk*. (E‐G) Histology of nLacZ^+^ structures. nLacZ^+^ cells in the proximal aorta (E), the sinoatrial node (F) and a cardiac ganglion (F). Scale bars 1 mm (A‐D), 10 μm (E), and 50 μm (F and G)

We first analyzed the effect of TB4 on the healthy myocardium in Isl1‐nLacZ (n = 3) and WT (n = 3) animals. Mice were treated with TB4 (12 mg/kg body weight, i.p.) over the course of 7 days. TB4 treatment had no effect on body weight, and the animals showed no obvious alteration in behavior (Figure 1). Although no nLacZ^+^ cells could be detected after treatment with TB4 in WT animals as expected, all Isl1‐nLacZ animals showed nLacZ^+^ cells in the 3 established distinct structures: the sinoatrial node, the outflow tract and parasympathetic ganglia in close proximity to the pulmonary veins (Figure 2). nLacZ^+^ cells could not be detected in the free ventricular wall of any of these animals (approximately 1200 sections analyzed, n = 6).

**Figure 3:**
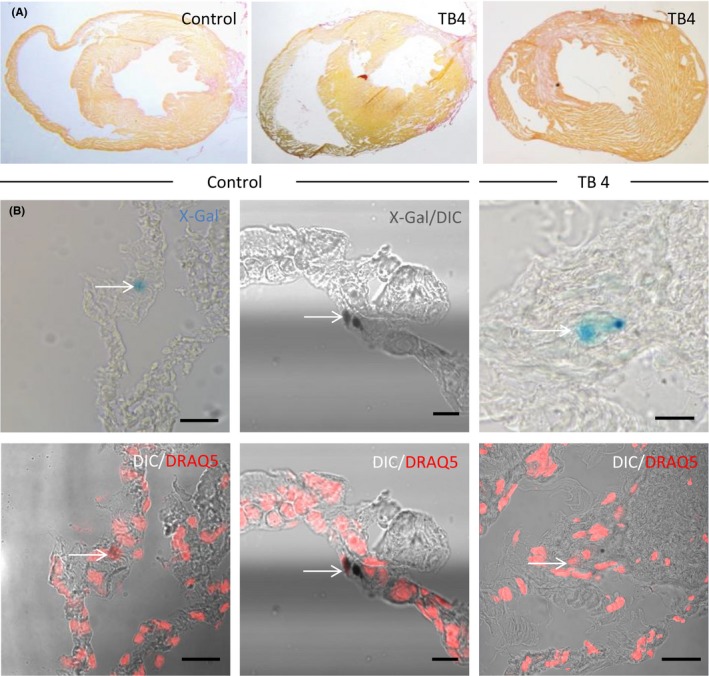
nLacZ‐positive cells in the infarct zone. (A) Cross sections of infarcted hearts from Isl1‐nLacZ animals 7 days after infarction. (B) nLacZ^+^ cells in the infarcted anterior wall of nLacZ control hearts (7 days after myocardial infarction and a TB4 primed heart (14 days after myocardial infarction). Visualization by bright‐field microscopy and differential interference contrast confocal microscopy (DIC)

**Figure 4:**
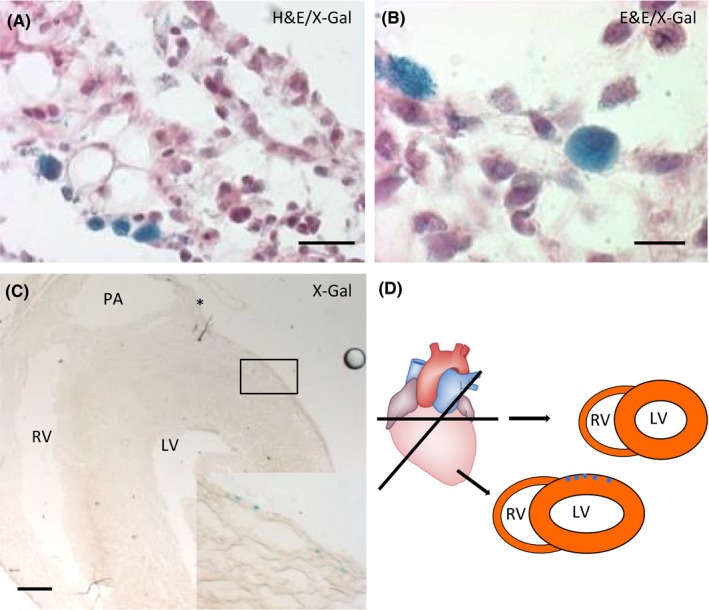
LacZ‐positive structures in infarcted hearts. (A and B) LacZ^+^ cells with a cytosolic staining pattern in the infarct region. (C and D) nLacZ^+^ cells in the outflow tract region that were initially classified as positive cells in the infarct region because of an oblique plane. Scale bars 50 μm (A), 10 340 μm (B), 500 μm (C)

We then evaluated the effect of TB4 on Islet‐1 expression after myocardial injury. LAD ligation was performed after 1 week of daily TB4 administration (12 mg/kg body weight per injection). Following LAD‐ligation TB4 was injected every other day. Hearts were harvested 2 (control n = 1; TB4 n = 2), 7 (control n = 3; TB4 n = 4) and 14 days (control n = 1; TB4 n = 2) after myocardial infarction (Figure 3A). Infarct size was small in TB4 primed hearts and control animals (4.74 ± 0.99% in PBS treated animals vs. 5.58 ± 1.38% in TB4 primed mice). Altogether, approximately 4000 tissue sections were analyzed for nLacZ^+^ structures. Again, we found positive cells in the sinoatrial node, the outflow tract, and cardiac ganglia. We identified 3 nLacZ^+^ cells that could not be ascribed to any of these 3 structures (Figure 3B): a single cell was detected in a TB4‐treated Isl1‐nLacZ mouse 14 days after LAD‐ligation. This cell was localized in an epicardial tissue adhesion. Costaining with DRAQ5 revealed a nuclear localization of the LacZ‐signal. Adjacent to that cell, a second blue signal suggested another nLacZ‐positive structure. However, this structure did not stain positive for DRAQ5 (Figure 3B). The other 2 nLacZ^+^ cells were detected in control‐injected Isl‐nLacZ mice 7 days after infarction (Figure 3B). DRAQ5 confirmed a nuclear localization of the LacZ‐signal in both cells. We initially considered more cells in the infarct region to be nLacZ^+^. However, a more rigorous evaluation revealed that those cells either showed a cytosolic LacZ‐staining pattern (Figure 4A and B) or were located in the outflow tract (Figure 4C and D).

### 3.2 TB4 effects in cardiac explant cultured

**Figure 5:**
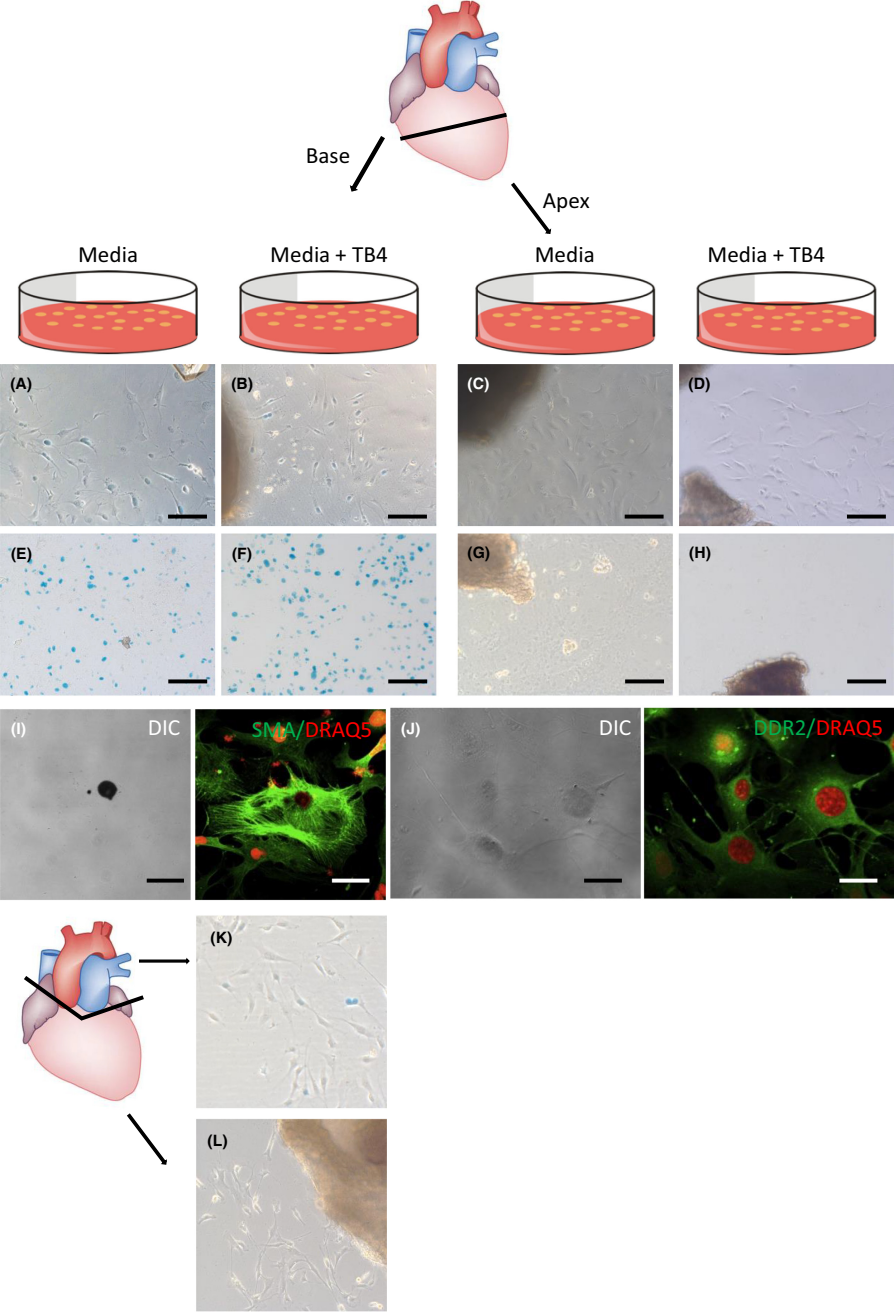
nLacZ‐positive cells in explant cultures. (A, B, E and F) nLacZ^+^ cells from the basal parts of the heart. (C, D, G and H) nLacZ^−^ cells from the apical parts of the heart. (I and J) nLacZ^+^ cells were positive for smooth muscle actin (SMA) and DDR2. (K) nLacZ^+^ cells in an explant culture from the outflow tract region. (L) nLacZ+ cells in an explant culture after dissection of the outflow tract region. Scale bars 500 μm (A, B, C, D), 1 mm (E, F, G, H), 40 μm (I), 20 μm (J)

It has been reported that TB4 upregulates Islet‐1 in explant cultures from the heart and eventually leads to the differentiation of epicardial cells to cardiomyocytes in vitro.^29^ We therefore also investigated the effect of TB4 on outgrowth cultures of heart pieces from Isl1‐nLacZ mice. Unexpectedly, we initially observed a high density of nLacZ^+^ cells, irrespective of the supplementation of TB4 in these explant cultures (n = 3 per group). To further understand the origin of these cells, we separated the heart in an Islet‐1^+^ basal part and an Islet‐1^−^ apical part and incubated pieces of them separately (n = 3 per group). While explant cultures from the basal part of the heart reliably contained nLacZ^+^ cells, explant cultures derived from the apical part never did (5A‐H), irrespective of TB4 treatment. Immunofluorescence labeling revealed that the nLacZ^+^ cells expressed smooth muscle actin (SMA) and discoidin domain‐containing receptor 2 (DDR2, Figure 5I and J). Dissection of the outflow tract area prior culture resulted in cell cultures that did not contain nLacZ^+^ cells in the basal part (Figure 5L, n = 2). Explant cultures derived from the outflow tract in contrast resulted in nLacZ^+^ cells (Figure 5K).

## 4 DISCUSSION

This study evaluated the effect of TB4 on the expression of the transcription factor Islet‐1 in a well‐characterized animal model that allows for an easy and unambiguous identification of Islet‐1^+^ cells. We identified only 1 single nLacZ^+^ cell outside of the already described Islet‐1^+^ regions in 8 infarcted hearts of TB4‐treated animals and 2 in 5 control animals that had not received TB4. Thus, our experiments failed to support previous studies suggesting that TB4 induces the expression of Islet‐1 in the ventricle after injury. They also did not provide evidence for a stimulatory effect of TB4 on Islet‐1 expression in cardiac explant cultures.

The apparent discrepancies with published work could have several reasons. (1) The heterozygous Isl‐nLacZ knock‐in model might not completely recapitulate the normal Islet‐1 expression (heteroinsufficiency). However, we and others have shown that this model reliably monitors Islet‐1 expression during development and adulthood.^3^, ^7^ Incomplete labeling in the knock‐in model might explain a lower than 100% sensitivity, but the complete absence of nLacZ^+^ cells in the infarct region argues against heteroinsufficiency as the cause of the discrepant results. (2) Our model does not allow for lineage tracing of Islet‐1^+^ cells. As Islet‐1 positivity represents only an intermediate phenotype we simply might have missed Islet‐1^+^ cells. We consider this possibility unlikely because we have used different time points. (3) The TB4 used in our in vivo and in vitro experiments may have been inactive or simply used incorrectly. We believe that we can exclude the latter as we strictly followed a protocol previously described by Smart et al. (2011) and, similar to their study, used clinical grade TB4 that was generously provided by RegeneRx Biopharmaceuticals. While no evidence for any other harm to the compound exists, we cannot finally rule out that anything was wrong with the lot of TB4. (4) Smart et al. have shown that the expression of Islet‐1 correlates with injury size and the infarcts in our study were relatively small. Yet, the complete absence of nLAcZ^+^ cells argues against this possibility. (5) TB4 does in fact not lead to an increase in Islet‐1 expression in the ventricle after injury. Detection of Islet‐1 with immunohistological methods is difficult and the use of high magnification complicates the analysis of the exact localization. The Isl1‐nLacZ model, in contrast, allows for unambiguous detection of positive and negative cells, leading us to have higher confidence in the method. The apparent absence of TB4 effects on the expression of Islet‐1 in explant cultures supports this notion. We only detected nLacZ^+^ cells in the explant cultures derived from parts of the heart that already contained Islet‐1^+^ cells, irrespective of TB4. These cells expressed the fibroblast markers SMA and DDR2. Our model does not allow lineage tracing and we therefore cannot rule out that these cells are (partially) derived from an Islet‐1^−^ population. The fact that nLacZ^+^ cells occurred only in explant cultures from the basal part of the heart indicates that these originated from preexisting Islet‐1^+^ smooth muscle cells and fibroblasts that we have previously described in the outflow tract.^7^ This assumption is further supported by the fact that dissection of the outflow tract prior to culture led to nLacZ^−^ cell cultures. The negative findings of this study are in line with results from other groups resulting in a controversial discussion of the role of TB4 in the heart.^17^, ^18^, ^30^, ^31^, ^32^, ^33^, ^34^ Interestingly there is conflict on its role during development as well as on its therapeutic potential. Our study focused on the expression of Islet‐1 after TB4 treatment and was not designed to evaluate de novo cardiomyogenesis or the therapeutic potential of TB4 in general. In particular the number of animals used in our study is too small to draw conclusions about functional effects or infarct size. Yet, our study is in agreement with a recent publication that did not detect an increase in Nkx2.5‐positive cells after TB4 treatment in injured hearts.^34^ The fact that we did not detect nLacZ^+^ cells in the ventricle supports our previous findings and suggests that possible regenerative effects of TB4 in the injured heart are independent of reexpression of the transcription factor Islet‐1.

## DISCLOSURE

None declared.

## References

[1] Roger VL . Heart failure compendium epidemiology of heart failure. Circ Res. 2013;240:646‐660. https://doi.org/10.1161/CIRCRESAHA.113.300268. 10.1161/CIRCRESAHA.113.300268 PMC3806290 23989710

[2] van Berlo JH , Molkentin JD . An emerging consensus on cardiac regeneration. Nat Med. 2014;242:1386‐1393. 10.1038/nm.3764 PMC4418535 25473919

[3] Sun Y , Liang X , Najafi N , et al. Islet 1 is expressed in distinct cardiovascular lineages, including pacemaker and coronary vascular cells. Dev Biol. 2007;304:286‐296. 17258700 10.1016/j.ydbio.2006.12.048 PMC2582044

[4] Moretti A , Caron L , Nakano A , et al. Multipotent embryonic isl1 + progenitor cells lead to cardiac, smooth muscle, and endothelial cell diversification. Cell. 2006;127:1151‐1165. 17123592 10.1016/j.cell.2006.10.029

[5] Laugwitz KL , Moretti A , Lam J , et al. Postnatal isl1 + cardioblasts enter fully differentiated cardiomyocyte lineages. Nature. 2005;433:647‐653. 15703750 10.1038/nature03215 PMC5578466

[6] Cai CL , Liang X , Shi Y , et al. Isl1 identifies a cardiac progenitor population that proliferates prior to differentiation and contributes a majority of cells to the heart. Dev Cell. 2003;5:877‐889. 14667410 10.1016/s1534-5807(03)00363-0 PMC5578462

[7] Weinberger F , Mehrkens D , Friedrich FW , Stubbendorff M , Hua X , Müller JC . Localization of islet‐1‐positive cells in the healthy and infarcted adult murine heart. Circ Res. 2012;110:1303‐1310.https://doi.org/10.1161/circresaha.111.259630 22427341 10.1161/CIRCRESAHA.111.259630 PMC5559221

[8] Khattar P , Friedrich FW , Bonne G , et al. Distinction between two populations of islet‐1‐positive cells in hearts of different murine strains. Stem Cells Dev. 2011;20:1043‐1052. 20942609 10.1089/scd.2010.0374 PMC5880329

[9] Vedantham V , Galang G , Evangelista M , Deo RC , Srivastava D . RNA sequencing of mouse sinoatrial node reveals an upstream regulatory role for Islet‐1 in cardiac pacemaker cells. Circ Res. 2015;116:797‐803. 25623957 10.1161/CIRCRESAHA.116.305913 PMC4344860

[10] Tessadori F , van Weerd JH , Burkhard SB , et al. Identification and functional characterization of cardiac pacemaker cells in zebrafish. PLoS ONE. 2012;7:e47644. 23077655 10.1371/journal.pone.0047644 PMC3473062

[11] Liang X , Zhang Q , Cattaneo P , et al. Transcription factor ISL1 is essential for pacemaker development and function. J Clin Invest. 2015;125:3256‐3268. 26193633 10.1172/JCI68257 PMC4563735

[12] Hoffmann S , Berger IM , Glaser A , Bacon C . 2013 Islet1 is a direct transcriptional target of the homeodomain transcription factor Shox2 and rescues the Shox2‐mediated bradycardia. Basic Res Cardiol. 108: 1‐11. https://doi.org/10.1007/s00395-013-0339-z 10.1007/s00395-013-0339-z PMC3597335 23455426

[13] Hannappel E , Xu G‐J , Morgan J , Hempstead J , Horecker BL . Thymosin beta 4: A ubiquitous peptide in rat and mouse‐tissues. Proc Natl Acad Sci USA. 1982;79:2171‐2175. 10.1073/pnas.79.7.2172 PMC346152 6954532

[14] Cassimeris L , Safer D , Nachmias VT , Zigmond SH . Thymosin beat 4 sequesters the majority of G‐actin in resting human polymorphonudear leukocytes. J Cell Biol. 1992;119:1261‐1270. 1447300 10.1083/jcb.119.5.1261 PMC2289708

[15] Bock‐Marquette I , Saxena A , White MD , Dimaio JM , Srivastava D . Thymosin beta4 activates integrin‐linked kinase and promotes cardiac cell migration, survival and cardiac repair. Nature. 2004;432:466‐472. 15565145 10.1038/nature03000

[16] Bock‐Marquette I , Shrivastava S , Pipes GT , et al. Thymosin beta4 mediated PKC activation is essential to initiate the embryonic coronary developmental program and epicardial progenitor cell activation in adult mice in vivo. J Mol Cell Cardiol. 2009;46:728‐738. 19358334 10.1016/j.yjmcc.2009.01.017 PMC2768533

[17] Banerjee I , Zhang J , Morris TM , et al. Thymosin beta 4 is dispensable for murine cardiac development and function. Circ Res. 2012;110:456‐464. https://doi.org/10.1161/circresaha.111.258616 22158707 10.1161/CIRCRESAHA.111.258616 PMC3739283

[18] Banerjee I , Morris TM , Evans SM , Chen J . Thymosin β4 is not required for embryonic viability or vascular development. Circ Res. 2013;112:e25‐e28. 23371905 10.1161/CIRCRESAHA.111.300197 PMC3712119

[19] Hinkel R , El‐Aouni C , Olson T , et al. Thymosin beta4 is an essential paracrine factor of embryonic endothelial progenitor cell‐mediated cardioprotection. Circulation. 2008;117:2232‐2240. 18427126 10.1161/CIRCULATIONAHA.107.758904 PMC2672916

[20] Chiu LLY , Reis LA , Radisic M . Controlled delivery of thymosin β4 for tissue engineering and cardiac regenerative medicine. Ann N Y Acad Sci. 2012;1269:16‐25. 23045966 10.1111/j.1749-6632.2012.06718.x

[21] Peng H , Xu J , Yang XP , et al. Thymosin‐β4 prevents cardiac rupture and improves cardiac function in mice with myocardial infarction. Am J Physiol Heart Circ Physiol. 2014;307:H741‐H751. 25015963 10.1152/ajpheart.00129.2014 PMC4187393

[22] Rui L , Yu N , Hong L , et al. Extending the time window of mammalian heart regeneration by thymosin beta 4. J Cell Mol Med. 2014;18:2417‐2424. 25284727 10.1111/jcmm.12421 PMC4302647

[23] Postrach J , Schmidt M , Thormann M , et al. Adeno‐associated viral vector 2.9 thymosin ß4 application attenuates rejection after heart transplantation: results of a preclinical study in the pig. Transplantation. 2014;98:835‐843. 25321165 10.1097/TP.0000000000000327

[24] Hinkel R , Ball HL , DiMaio JM , et al. C‐terminal variable AGES domain of Thymosin β4: The molecule's primary contribution in support of post‐ischemic cardiac function and repair. J Mol Cell Cardiol. 2015;87:113‐125. 26255251 10.1016/j.yjmcc.2015.07.004

[25] Hinkel R , Howe A , Renner S , et al. Diabetes mellitus‐induced microvascular destabilization in the myocardium. J Am Coll Cardiol. 2017;69:131‐143. 28081822 10.1016/j.jacc.2016.10.058

[26] Qian L , Huang Y , Spencer CI , et al. In vivo reprogramming of murine cardiac fibroblasts into induced cardiomyocytes. Nature. 2012;485:593‐598. 22522929 10.1038/nature11044 PMC3369107

[27] Smart N , Bollini S , Dubé KN , et al. De novo cardiomyocytes from within the activated adult heart after injury. Nature. 2011;474:640‐644. 21654746 10.1038/nature10188 PMC3696525

[28] Kolk MV , Meyberg D , Deuse T , et al. LAD‐ligation: A murine model of myocardial infarction. J Vis Exp. 2009;. https://doi.org/10.3791/1438. 10.3791/1438 PMC3164062 19829290

[29] Smart N , Riley PR . Derivation of epicardium‐derived progenitor cells (EPDCs) from adult epicardium. In: 000 00, eds. Current Protocols in Stem Cell Biology. Hoboken, NJ: John Wiley & Sons, Inc.; 2009 https://doi.org/10.1002/9780470151808.sc02c02s8 10.1002/9780470151808.sc02c02s8 19235142

[30] Rudat C , Kispert A . Wt1 and epicardial fate mapping. Circ Res. 2012;111:165‐169. https://doi.org/10.1161/circresaha.112.273946 22693350 10.1161/CIRCRESAHA.112.273946

[31] Rossdeutsch A , Smart N , Dubé KN , Turner M , Riley PR . Essential role for thymosin β4 in regulating vascular smooth muscle cell development and vessel wall stability. Circ Res. 2012;111:e89‐e102.https://doi.org/10.1161/circresaha.111.259846 22723298 10.1161/CIRCRESAHA.111.259846

[32] Smart N , Riley PR . Thymosin β4 in vascular development response to research commentary. Circ Res. 2013;112:e29‐e30. 23371906 10.1161/CIRCRESAHA.112.300555 PMC3978174

[33] Stark CK , Tarkia M , Kentala R , et al. Systemic dosing of thymosin beta 4 before and after ischemia does not attenuate global myocardial ischemia‐reperfusion injury in pigs. Front Pharmacol. 2016;7:115. 27199757 10.3389/fphar.2016.00115 PMC4853610

[34] Deutsch MA , Doppler SA , Li X , et al. Reactivation of the Nkx2.5 cardiac enhancer after myocardial infarction does not presage myogenesis. Cardiovasc Res. 2018;324:98 https://doi.org/10.1093/cvr/cvy069. 10.1093/cvr/cvy069 PMC6279078 29579159

